# Divergent age-related cognitive impairments in first-episode psychosis

**DOI:** 10.1016/j.scog.2025.100394

**Published:** 2025-09-22

**Authors:** ChengFei Duan, ChunHua Cao, MingLiang Ju, YanYan Wei, XiaoChen Tang, LiHua Xu, HuiRu Cui, YingYing Tang, ZhengHui Yi, Xin Wei, JiJun Wang, TianHong Zhang

**Affiliations:** a Department of Psychiatry, The Eighth People's Hospital of Zhengzhou, Zhengzhou, China; b Shanghai Sixth People's Hospital Affiliated to Shanghai Jiao Tong University School of Medicine, Shanghai, China; c Shanghai Mental Health Center, Shanghai Jiaotong University School of Medicine, Shanghai Engineering Research Center of Intelligent Psychological Evaluation and Intervention, Neuroregulation Center, Shanghai, 200030, China; d The Brain Hospital of Guangxi Zhuang Autonomous Region, LiuZhou, China; e Department of Psychiatry, Nantong Fourth People's Hospital & Nantong Brain Hospital, Suzhou, 226000, China

**Keywords:** Psychosis, Neurocognition, Age, First-episode, Schizophrenia

## Abstract

**Background:**

Cognitive impairment is a core feature of first-episode psychosis (FEP), but its age-associated cognitive patterns remain unclear. Prior studies suggest FEP is associated with baseline cognitive deficits and accelerated decline, yet inconsistencies exist regarding whether cognitive aging in FEP mirrors or diverges from healthy aging.

**Methods:**

We compared 378 drug-naive FEP patients and 477 healthy controls (HC) using the Measurement and Treatment Research to Improve Cognition in Schizophrenia (MATRICS) Consensus Cognitive Battery (MCCB). Clinical symptoms were evaluated via the Positive and Negative Syndrome Scale (PANSS). Age-correlations with cognitive domains were analyzed via Pearson's coefficients and Fisher's z-transformation.

**Results:**

FEP patients showed significant deficits across all cognitive domains (all *p* < 0.001) and disrupted age-associated cognitive patterns. In HC, age was associated with gradual declines in memory (e.g., HVLT-R *r* = −0.304, *p* < 0.001), working memory (*r* = −0.168, *p* < 0.001), and learning functions, aligning with normative aging. FEP patients showed a complex pattern: while some executive functions (e.g., Trail Making A) mirrored HC's negative age correlations, social cognition (*r* = 0.174, *p* < 0.001), attention (*r* = 0.125, *p* = 0.015), and specific learning domains exhibited positive age associations. Group comparisons revealed significant differences in age-cognition relationships for verbal memory, working memory, and overall cognitive composites (all *p* < 0.0028 after Bonferroni correction), indicating disrupted cognitive aging in FEP.

**Conclusions:**

FEP disrupts normative cognitive aging patterns, characterized by atypical decline and compensatory improvements. These findings highlight the need for longitudinal studies to clarify mechanisms and inform age-adapted interventions.

## 1 Introduction

Cognitive impairment is a well-documented feature in individuals experiencing their first episode of psychosis (FEP) (Amoretti et al., 2021; Cohen et al., 2025; Millgate et al., 2023). A systematic review and meta-analysis has revealed that antipsychotic drug-naive patients with FEP exhibit significant cognitive impairments across all domains (speed of processing, verbal/visual learning, working memory, attention, reasoning/problem solving, executive function) compared to healthy controls, with large effect sizes (Hedges g = −1.16 to −0.68), and display heightened within-group variability in cognitive performance, underscoring the need to characterize heterogeneity in early-stage psychosis (Lee et al., 2024). Emerging evidence indicates that these cognitive deficits may precede the onset of full-blown psychosis, as demonstrated in studies on premorbid functioning in FEP patients. For example, Cohen et al. conducted a meta-analysis and found that individuals who later developed schizophrenia exhibited consistent cognitive impairments in verbal memory, working memory, and processing speed during the premorbid phase (i.e., before the first psychotic episode), with these deficits persisting and worsening after illness onset (Cohen et al., 2025). Another study by Sponheim et al. compared cognitive performance between recent-onset FEP patients and healthy controls, and further traced back to premorbid educational and cognitive assessments; their results showed that FEP patients had significantly lower premorbid cognitive scores (especially in attention and executive function) than controls, confirming that cognitive deficits are not a consequence of illness but predate the first episode (Sponheim et al., 2010). These findings highlight the importance of early cognitive assessment in FEP, as they imply that cognitive impairment is not merely a consequence of chronic illness but may represent a core feature from the disorder's inception.

While premorbid cognitive deficits in FEP patients directly confirm the early emergence of cognitive impairment before psychosis onset, similar patterns of cognitive vulnerability have also been observed in individuals at clinical high risk for psychosis (CHR-P)—a population that has not yet developed full-blown psychotic symptoms but faces elevated risk of transition to psychosis. As demonstrated in prior studies, CHR-P individuals exhibit notable cognitive differences compared to healthy controls, providing further context for understanding the continuity of cognitive dysfunction across the psychosis spectrum (Hui et al., 2014; Seidman et al., 2016; Zhang et al., 2015, Zhang et al., 2024b). Another systematic review and meta-analysis has shown that individuals at clinical high risk for psychosis (CHR-P) exhibit moderate to large neurocognitive deficits compared to healthy controls in domains such as processing speed (e.g., digit symbol coding test, g = −0.74), verbal learning (e.g., Hopkins Verbal Learning Test-Revised, g = −0.86), and executive function (e.g., Stroop color word reading task, g = −1.17), are less impaired than those with first-episode psychosis, and demonstrate greater deficits in longitudinal transition to psychosis, highlighting neurocognitive dysfunction as a potential biomarker for detection and prognosis (Catalan et al., 2021). These findings highlight the importance of early cognitive assessment in FEP, as they imply that cognitive impairment is not merely a consequence of chronic illness but may represent a core feature from the disorder's inception.

Despite the established presence of cognitive deficits in FEP (Haring et al., 2017; Sang et al., 2025), the age-associated cognitive patterns of these impairments remain poorly understood. Previous research has yielded conflicting results: some studies suggest accelerated cognitive aging in schizophrenia, with patients exhibiting cognitive performance comparable to healthy individuals several decades older (Harvey and Rosenthal, 2018), while others argue that cognitive decline in schizophrenia follows an age-associated patterns similar to normal aging but from a lower baseline (Valsdottir et al., 2020). Further research has revealed that individuals with early-onset first-episode psychosis (≤18 years) exhibit more profound global cognitive impairment, executive dysfunction, and sustained attention deficits compared to those with youth (19–24 years) or adult (≥25 years) onset, with no significant cognitive differences observed between youth and adult onset groups (De la Serna et al., 2023). These discrepancies may stem from differences in sample characteristics, such as disease stage or comorbid factors, underscoring the need for more targeted investigations in drug-naive FEP populations.

Against this backdrop, the present study aims to characterize age-related cognitive impairments in drug-naive individuals with FEP and compare them to healthy controls (HC). We hypothesize that FEP is associated with disrupted cognitive aging patterns, manifesting as atypical declines in specific cognitive domains. By employing a comprehensive neurocognitive battery, we seek to clarify whether cognitive aging in FEP is marked by accelerated decline, altered age-associated pattern, or a combination of both, providing critical insights into the age-adapted intervention strategies.

## 2 Methods

### 2.1 Study cohort and data collection

Baseline clinical and cognitive data were derived from a national study (National Key R&D Program of China, 2016YFC1306800) (Hu et al., 2025; Zhang et al., 2023b, Zhang et al., 2024a), conducted between 2016 and 2021, focusing on identifying biological markers for psychosis staging and developing early intervention strategies. The sample included 378 individuals with FEP and 477 healthy controls (HC) recruited at the Shanghai Mental Health Centre (SMHC). All FEP participants met criteria for non-affective first-episode psychosis (schizophrenia, schizophreniform disorder, or brief psychotic disorder) based on the Diagnostic and Statistical Manual of Mental Disorders, Fourth Edition, Text Revision (DSM-IV-TR), with no prior exposure to psychotropic medications. HC were free of current or lifetime psychiatric disorders, as confirmed by structured clinical interviews. Exclusion criteria for both groups included history of substance abuse/dependence, neurological diseases, or significant medical comorbidities.

Ethical approval was obtained from the Research Ethics Committee of SMHC (IRB2016-009), with adherence to the 2008 revised Declaration of Helsinki. Written informed consent was obtained from all participants after detailed explanation of study procedures. Data collection focused on demographic characteristics, clinical assessments (including Positive and Negative Syndrome Scale [PANSS] for FEP), and comprehensive neurocognitive testing, with all procedures reviewed and approved by relevant institutional ethics committees.

### 2.2 Measurements

The clinical psychopathology of FEP individuals was evaluated using the PANSS (Kay et al., 1987). The PANSS comprises 30 items categorized into three subscales: positive symptoms, negative symptoms, and general psychopathology. Each item is rated on a 7-point Likert scale (1 = absent to 7 = extreme severity). Structured clinical interviews were conducted by two senior psychiatrists who had undergone specialized training and certification in standardized PANSS administration for research purposes.

Cognitive functions were evaluated using the Chinese version of the Measurement and Treatment Research to Improve Cognition in Schizophrenia (MATRICS) Consensus Cognitive Battery (MCCB), which has been validated for use in Chinese populations with psychosis (Kern et al., 2011, Kern et al., 2008). Trained examiners administered the battery following strict standardized procedures detailed in the MCCB manual to ensure reliability and consistency across participants. The assessment included nine subtests: Trail Making Test Part A (TMT-A), Brief Assessment of Cognition in Schizophrenia (BACS) Symbol Coding, Category Fluency Test, Continuous Performance Test–Identical Pairs (CPT-IP), Wechsler Memory Scale–Third Edition (WMS-3) Spatial Span, Hopkins Verbal Learning Test–Revised (HVLT-R), Brief Visuospatial Memory Test–Revised (BVMT-R), Mayer-Salovey-Caruso Emotional Intelligence Test (MSCEIT), and the Neuropsychological Assessment Battery (NAB) Mazes. Previous research on Chinese individuals with psychosis reported test–retest reliability coefficients ranging from 0.73 to 0.94 for these subtests (Shi et al., 2013). Collectively, these subtests comprehensively assess six key neurocognitive domains: speed of processing, attention/vigilance, working memory, verbal learning, visual learning, reasoning and problem solving, as well as social cognition.

### 2.3 Statistical analysis

**Fig. 1:**
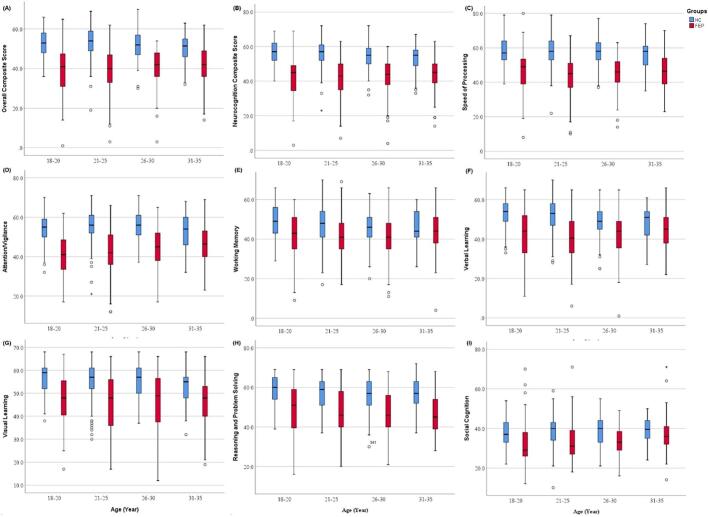
Age-associated cognitive performance across domains in HC and FEP groups. Box plots illustrating the distribution of cognitive composite scores across different age bands (18–20, 21–25, 26–30, 31–35 years) for healthy controls (HC, blue) and first-episode psychosis (FEP, red) groups. Panels represent: (A) Overall composite score; (B) neurocognitive composite score; (C) speed of processing; (D) attention/vigilance; (E) working memory; (F) verbal learning; (G) visual learning; (H) reasoning and problem solving; (I) social cognition. Note: The box represents the interquartile range (25th to 75th percentile), the line within the box is the median, and whiskers extend to 1.5 times the interquartile range. Outliers are plotted as individual points.

**Fig. 2:**
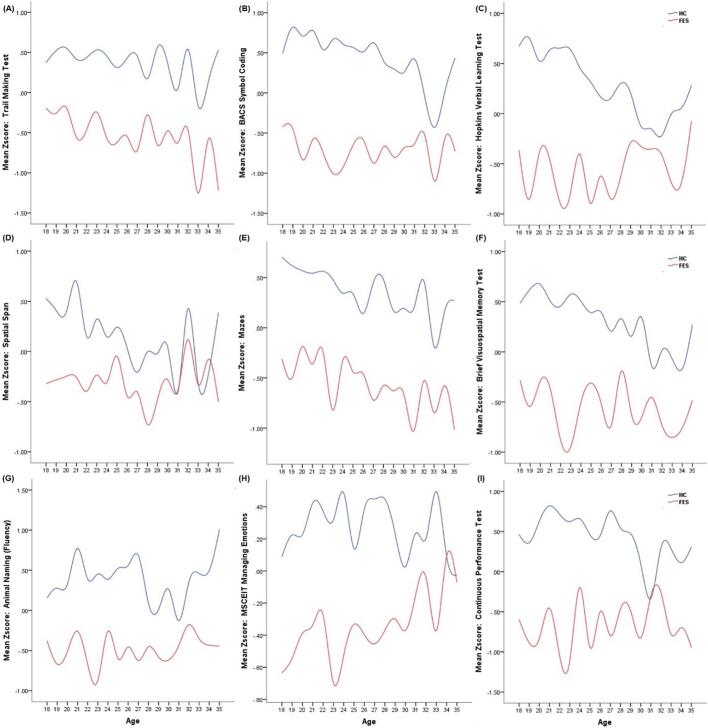
Age-associated patterns of cognitive performance in healthy controls (HC) and first-episode psychosis (FEP). Mean z-scores of cognitive tests are plotted against age for HC (blue line) and FEP (red line). Panels correspond to: (A) Trail Making Test A; (B) Brief Assessment of Cognition in Schizophrenia (BACS) Symbol Coding; (C) Hopkins Verbal Learning Test–Revised (HVLT-R); (D) Wechsler Memory Scale–Third Edition Spatial Span; (E) Neuropsychological Assessment Battery (NAB) Mazes; (F) Brief Visuospatial Memory Test–Revised (BVMT-R); (G) Category Fluency (Animal Naming); (H) Mayer-Salovey-Caruso Emotional Intelligence Test (MSCEIT; Managing Emotions); (I) Continuous Performance Test–Identical Pairs (CPT-IP). Note: Z-scores were computed using the entire sample. Given that higher raw scores on the Trail Making Test A (TMT-A) indicate poorer cognitive performance (contrary to other tests where higher scores reflect better performance), TMT-A z-scores were negated to ensure consistent interpretability (higher z-scores signify better performance across all tests).

**Fig. 3:**
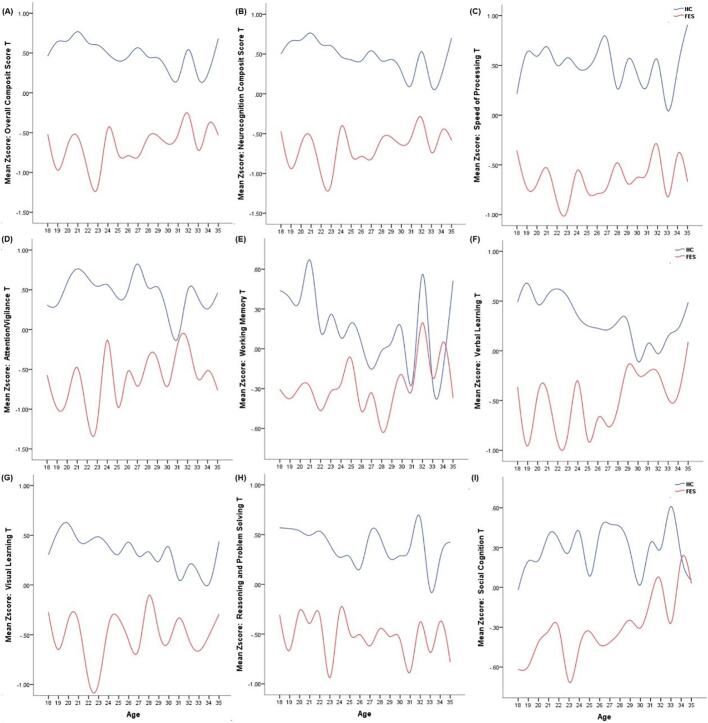
Age-associated patterns of composite cognitive performance in healthy controls (HC, blue line) and first-episode psychosis (FEP, red line). Mean z-scores for each cognitive domain are plotted against age. Panels correspond to: (A) Overall composite score; (B) neurocognition composite score; (C) speed of processing; (D) attention/vigilance; (E) working memory; (F) verbal learning; (G) visual learning; (H) reasoning and problem solving; (I) social cognition. Note: Z-scores were computed using the entire sample.

Group differences in demographic, clinical, and neurocognitive variables (Table 1) were assessed using independent-samples *t*-tests for continuous variables (e.g., age, education) and Pearson's chi-square test for categorical variables (gender). Box plots were generated to visualize cognitive performance across different age bands (18–20, 21–25, 26–30, 31–35 years) for HC and FEP groups across various cognitive domains (Fig. 1). For neurocognitive performance patterns (Fig. 2, Fig. 3), z-scores were computed for all tests using the entire sample (HC + FEP) to standardize performance; notably, TMT-A z-scores were negated (since higher raw TMT-A scores indicate poorer cognition) to align with the interpretive direction of other tests (higher z-scores = better performance). Correlations between age and neurocognitive functions (Table 2) were calculated via Pearson's correlation coefficients for HC and FEP separately. To compare age-cognition correlation strengths between groups (Table 3), Fisher's z-transformation converted Pearson r values to normally distributed z scores, enabling group-level comparisons. Multiple comparisons were corrected using the Bonferroni method (α = 0.05/18 ≈ 0.0028) to account for 18 cognitive variables. Age-related performance trends in Fig. 1, Fig. 2 were visualized by plotting mean z-scores against age for HC and FEP, illustrating group differences in age-associated cognitive patterns. Sample sizes were *n* = 477 (HC) and *n* = 378 (FEP) across all analyses. To formally test age-associated cognitive trends, separate analysis of covariance (ANCOVA) with polynomial contrasts was conducted for HC and FEP groups, controlling for gender and education. Both linear and quadratic trends in the relationship between age and overall cognitive composite score were examined.

**Table 1:** Demographic, clinical, and cognitive characteristics of healthy controls (HC) and first-episode psychosis (FEP) groups.

Variables [mean/S.D.]	HC (N = 477)		FEP (N = 378)		Comparison	
	Mean/n	S.D./%	Mean/n	S.D./%	*t*/***χ***^***2***^	*P* value
Age (years)	23.84	4.13	25.82	5.22	−6.180	<0.001
Male [n (%)]	267	56.0 %	210	55.6 %	***χ***^***2***^ = 0.015	0.902
Female [n (%)]	210	44.0 %	168	44.4 %		
Education (years)	14.92	2.68	12.94	3.09	10.041	<0.001
Father education (years)	11.09	3.47	10.02	3.68	3.744	<0.001
Mother education (years)	10.25	3.76	8.86	3.98	4.442	<0.001
PANSS-P	–	–	21.57	5.96	–	–
PANSS-N	–	–	17.45	7.16	–	–
PANSS-G	–	–	39.31	7.93	–	–
PANSS-Total	–	–	78.31	16.07	–	–
Trail Making A	27.61	9.68	44.50	21.47	−15.336	<0.001
BACS symbol coding	65.03	10.11	48.21	11.52	22.718	<0.001
HVLT-R	26.85	4.32	21.33	5.60	16.284	<0.001
WMS-3 spatial span	16.53	2.88	14.77	3.37	8.262	<0.001
NAB mazes	19.54	4.89	13.17	6.66	16.101	<0.001
BVMT-R	28.65	5.09	21.56	7.69	16.145	<0.001
Category Fluency	24.11	5.68	18.86	5.43	13.689	<0.001
MSCEIT	87.04	8.45	80.70	10.40	9.832	<0.001
CPT-IP	2.99	0.61	2.01	0.78	20.686	<0.001
Speed of processing	57.85	8.12	45.08	10.83	19.699	<0.001
Attention/vigilance	55.20	7.53	43.72	10.37	18.717	<0.001
Working memory	47.52	9.23	42.37	10.74	7.534	<0.001
Verbal learning	51.47	8.01	42.07	11.30	14.208	<0.001
Visual learning	55.97	7.54	46.75	11.44	14.155	<0.001
Reasoning and problem solving	57.26	7.95	47.65	11.11	14.730	<0.001
Social cognition	38.62	6.78	33.97	8.41	8.933	<0.001
Overall composite score	52.70	7.11	40.14	10.54	20.756	<0.001
Neurocognition composite score	55.47	7.15	42.75	10.69	20.769	<0.001

Note. Continuous variables are reported as mean ± standard deviation (S.D.), and categorical variables (gender) as frequency (percentage). Positive and Negative Syndrome Scale (PANSS) scores were only calculated for the FEP group, as HC had no psychotic symptomology. Abbreviations: BACS, Brief Assessment of Cognition in Schizophrenia symbol coding; BVMT-R, Brief Visuospatial Memory Test–Revised; CPT-IP, Continuous Performance Test–Identical Pairs; HVLT-R, Hopkins Verbal Learning Test–Revised; NAB, Neuropsychological Assessment Battery mazes; WMS-3, Wechsler Memory Scale–Third Edition spatial span. MSCEIT, Mayer-Salovey-Caruso Emotional Intelligence Test.

**Table 2:** Correlation between age and cognitive functions (within-group correlations).

Cognitive variables	Overall		HC		FEP	
	*r*	*p*	*r*	*p*	*r*	*p*
Trail Making A	0.218**	<0.001	0.117*	0.011	0.159**	0.002
BACS symbol coding	−0.227**	<0.001	−0.218**	<0.001	−0.053	0.307
HVLT-R	−0.187**	<0.001	−0.304**	<0.001	0.056	0.280
WMS-3 spatial span	−0.149**	<0.001	−0.220**	<0.001	0.004	0.932
NAB mazes	−0.259**	<0.001	−0.210**	<0.001	−0.168**	0.001
BVMT-R	−0.215**	<0.001	−0.226**	<0.001	−0.075	0.146
Category fluency	−0.075*	0.028	−0.007	0.886	0.037	0.473
MSCEIT	0.011	0.738	0.016	0.725	0.138**	0.007
CPT-IP	−0.140**	<0.001	−0.126**	0.006	0.053	0.302
Speed of processing	−0.114**	0.001	−0.019	0.683	0.019	0.716
Attention/vigilance	−0.062	0.068	−0.030	0.507	0.125*	0.015
Working memory	−0.095**	0.006	−0.168**	<0.001	0.060	0.247
Verbal learning	−0.099**	0.004	−0.207**	<0.001	0.129*	0.012
Visual learning	−0.126**	<0.001	−0.112*	0.014	0.006	0.910
Reasoning and problem solving	−0.174**	<0.001	−0.121**	0.008	−0.072	0.165
Social cognition	0.056	0.101	0.064	0.165	0.174**	0.001
Overall composite score	−0.121**	<0.001	−0.135**	0.003	0.089	0.083
Neurocognition composite score	−0.141**	<0.001	−0.160**	<0.001	0.064	0.216

Note: Abbreviations: BACS, Brief Assessment of Cognition in Schizophrenia symbol coding; BVMT-R, Brief Visuospatial Memory Test–Revised; CPT-IP, Continuous Performance Test–Identical Pairs; HVLT-R, Hopkins Verbal Learning Test–Revised; NAB, Neuropsychological Assessment Battery mazes; WMS-3, Wechsler Memory Scale–Third Edition spatial span. MSCEIT, Mayer-Salovey-Caruso Emotional Intelligence Test.

* p < 0.05.

** p < 0.01.

**Table 3:** Comparison of age-cognition correlation coefficients (HC vs. FEP) using Fisher's z-transformation (between-group comparisons).

Cognitive variables	*Z*_*HC*_	*Z*_*FEP*_	*S.E.*	*Z* score	*p*	Sig. (corrected) (*p* < 0.0028)
Trail Making A	0.118	0.161	0.069	0.623	0.533	No
BACS symbol coding	−0.227	−0.053	0.069	−2.522	0.012	No
HVLT-R	−0.33	0.056	0.069	5.609	<0.001	Yes
WMS-3 spatial span	−0.229	0.004	0.069	3.406	0.0006	Yes
NAB mazes	−0.219	−0.172	0.069	−0.681	0.496	No
BVMT-R	−0.237	−0.076	0.069	−2.333	0.02	No
Category fluency	−0.007	0.037	0.069	0.638	0.524	No
MSCEIT	0.016	0.14	0.069	1.794	0.073	No
CPT-IP	−0.127	0.053	0.069	2.609	0.009	No
Speed of processing	−0.019	0.019	0.069	0.551	0.582	No
Attention/vigilance	−0.03	0.127	0.069	2.275	0.023	No
Working memory	−0.175	0.061	0.069	3.42	0.0006	Yes
Verbal learning	−0.218	0.131	0.069	5.058	<0.001	Yes
Visual learning	−0.113	0.006	0.069	1.725	0.084	No
Reasoning and problem solving	−0.122	−0.073	0.069	0.71	0.478	No
Social cognition	0.064	0.178	0.069	1.652	0.098	No
Overall composite score	−0.137	0.09	0.069	3.29	0.001	Yes
Neurocognition composite score	−0.164	0.065	0.069	3.319	0.001	Yes

Note: Statistical methods: Fisher's z-transformation was used to convert Pearson correlation coefficients (r) to normally distributed z values, enabling group comparisons. The formula for z-transformation is (z = 0.5 × ln[(1 + *r*)/(1 − *r*)]). The standard error (S.E.) was calculated as, $\sqrt{1/\left(n_{1}-3\right)+1/\left(n_{2}-3\right)}$ where n_HC_ = 477 and n_FEP_ = 378. The z statistic reflects the difference between z-transformed values of HC and FEP, with significance determined by a two-tailed test.

Multiple comparisons correction: Bonferroni correction was applied (significance threshold alpha = 0.05/18 (0.0028)) for statistical significance.

Abbreviations: BACS: Brief Assessment of Cognition in Schizophrenia (symbol coding subtest); BVMT-R: Brief Visuospatial Memory Test–Revised; CPT-IP: Continuous Performance Test–Identical Pairs; HVLT-R: Hopkins Verbal Learning Test–Revised; NAB: Neuropsychological Assessment Battery (mazes subtest); WMS-3: Wechsler Memory Scale–Third Edition (spatial span subtest); MSCEIT: Mayer-Salovey-Caruso Emotional Intelligence Test; SE: standard error.

## 3 Results

### 3.1 Demographic, clinical and cognitive characteristics

Table 1 compares demographic, clinical, and neurocognitive profiles between HC (*N* = 477) and FEP (*N* = 378). Demographically, FEP participants were older (*M* = 25.82 vs. 23.84 years, *p* < 0.001) but had lower educational attainment (*M* = 12.94 vs. 14.92 years, *p* < 0.001), along with reduced parental education (both *p* < 0.001). Gender distribution was equivalent across groups (*p* = 0.902). Cognitively, FEP performed significantly worse than HC across all assessed domains (all *p* < 0.001), including executive function (e.g., Trail Making A: *M* = 44.50 vs. 27.61), memory (e.g., HVLT-R: *M* = 21.33 vs. 26.85), social cognition (*M* = 33.97 vs. 38.62), and both composite scores (Overall: *M* = 40.14 vs. 52.70; Neurocognition: *M* = 42.75 vs. 55.47).

### 3.2 Cognitive performance by age and group

Fig. 1 presents age-associated cognitive performance across various domains in HC and FEP groups, with participants grouped into age bands of 18–20, 21–25, 26–30, and 31–35 years. HC consistently achieved higher scores than FEP in all domains, including overall composite, neurocognitive composite, speed of processing, attention/vigilance, working memory, verbal learning, visual learning, reasoning and problem solving, and social cognition.

### 3.3 Age-associated cognitive patterns in HC and FEP

Fig. 2 illustrates age-associated cognitive patterns (mean z-scores) in HC (blue) and FEP (red) across nine tests. In memory domains, HC exhibited more consistent age-related declines: for example, HVLT-R (Panel C) and WMS-3 Spatial Span (Panel D) showed steady z-score reductions with aging, whereas FEP displayed erratic fluctuations. Executive function tasks (Trail Making A, Panel A; NAB Mazes, Panel E) revealed more stable age trends in HC versus pronounced volatility in FEP. Notably, social-emotional cognition (MSCEIT Managing Emotions, Panel H) showed reversed age-correlated patterns, with FEP z-scores trending upward as age increased, contrasting HC's downward age-associated pattern. Overall, FEP demonstrated disrupted age-cognitive performance profiles compared to HC, particularly in memory and social-emotional domains.

Fig. 3 illustrates age-associated performance patterns (mean z-scores) of composite and domain-specific cognitive functions in HC (blue) and FEP (red). The Overall Composite Score (Panel A) and Neurocognition Composite Score (Panel B) showed stable age-associated declines in HC, whereas FEP exhibited marked fluctuations and occasional reversed trends. In domain-specific analyses, HC demonstrated consistent age-related decreases in Working Memory (Panel E) and Verbal Learning (Panel F), while FEP displayed erratic oscillations. Notably, Social Cognition (Panel I) revealed an upward age trend in FEP, contrasting with HC's downward trajectory. Across composite scores and most domains, FEP exhibited disrupted age-cognition profiles, with particularly pronounced irregularities in Working Memory, Verbal Learning, and Social Cognition.

### 3.4 Age-cognition correlation patterns in HC and FEP

In Table 2, age showed significant positive correlations with Trail Making A in all groups. Negative age-correlations were prominent in HC for memory (HVLT-R, WMS-3 spatial span), working memory, and verbal/visual learning (all *p* < 0.05), while FEP exhibited reversed patterns: positive correlations in MSCEIT, Attention/Vigilance, Verbal Learning, and Social Cognition (*p* < 0.05), with only NAB mazes showing significant negative correlation. Overall and Neurocognition Composite Scores correlated negatively with age in HC but not in FEP.

Separate ANCOVA with polynomial contrasts, controlling for gender and education, revealed distinct age-associated cognitive patterns between HC and FEP. In HC, there was a significant negative linear relationship between age and overall cognitive composite score (F = 27.785, *p* < 0.001, partial η^2^ = 0.055), with no significant quadratic trend (F = 1.210, *p* = 0.271). In contrast, FEP showed no significant linear (F = 0.598, *p* = 0.440) or quadratic (F = 0.876, *p* = 0.350) age-related trends in overall cognitive composite score. Education had a significant positive effect on overall cognitive composite score in both groups (HC: F = 36.735, p < 0.001; FEP: F = 51.690, p < 0.001), while gender exhibited a significant negative effect only in HC (F = 6.939, *p* = 0.009) and not in FEP (F = 0.281, *p* = 0.597). The overall models were significant for both groups (HC: F = 17.176, p < 0.001, adjusted R^2^ = 0.089; FEP: F = 18.581, p < 0.001, adjusted R^2^ = 0.119).

### 3.5 Group differences in age-cognition association patterns

In Table 3, we compared age-cognition correlation patterns between HC (*n* = 477) and FEP (*n* = 378) using Fisher's z-transformation. Notably, FEP showed significantly different age-cognition associations compared to HC in several key domains: HVLT-R (verbal memory), WMS-3 spatial span, working memory, verbal learning, and overall/neurocognition composite scores (all *p* < 0.0028 after Bonferroni correction). For example, HC exhibited strong negative age-correlations in the HVLT-R (z = −0.33), whereas FEP showed a positive trend (z = 0.056), indicating disrupted memory aging trajectories in psychosis. Similarly, working memory and verbal learning correlations with age were reversed in FEP, while composite scores revealed disrupted overall cognitive aging patterns. In contrast, executive function tasks like Trail Making A and NAB mazes showed no significant group differences.

## 4 Discussion

The study reveals divergent age-associated cognitive patterns between HC and individuals with FEP, indicating that early psychosis disrupts typical cognitive aging patterns. In HC, age was generally associated with gradual declines in memory, working memory, and learning functions, consistent with normative cognitive aging. Conversely, FEP showed a complex pattern: while some executive functions mirrored HC's negative age correlations, social cognition, attention, and certain learning domains exhibited positive associations with age, suggesting atypical cognitive changes over time (Malliaris et al., 2024). Group comparisons further demonstrated significant differences in age-cognition relationships, particularly in verbal memory, working memory, and overall cognitive composites. These findings imply that FEP may dysregulate the normal interplay between aging and cognitive function, potentially due to altered neural plasticity, accelerated neurodegeneration, or disrupted compensatory mechanisms (Gaweda, 2015; Zhang et al., 2016, Zhang et al., 2018).

The divergent age-associated cognitive patterns between HC and FEP likely reflect three interrelated clinical and sample-driven factors. First, the onset of first-episode psychosis may directly disrupt normal cognitive aging patterns by interfering with daily cognitive demands and functional neural networks (Kim et al., 2023). Psychotic symptoms (e.g., delusions, hallucinations) could exacerbate cognitive load, leading to atypical performance declines or compensatory strategies that distort age-related trends. For instance, the paradoxical positive age-correlations observed in FEP's social cognition might reflect learned adaptive behaviors in response to persistent social dysfunction, rather than normative developmental trajectories (Nour et al., 2025).

Second, baseline cognitive deficits in FEP could shape distinct aging trajectories independent of biological mechanisms. Compared to HC, FEP individuals exhibit preexisting impairments across memory, attention, and executive domains, which may create “floor effects” that mask typical age-related decline or produce atypical patterns (Jeste et al., 2011). This aligns with Valsdottir et al., who found that cognitive decline in schizophrenia follows a similar trajectory to healthy controls, with older patients' deficits primarily explained by lower baseline cognitive function rather than accelerated aging (Valsdottir et al., 2020). Reduced cognitive reserve in FEP might also limit the brain's capacity to maintain function with age, leading to steeper declines in some domains (e.g., verbal memory) or apparent stability in others due to ceiling effects (Harvey and Rosenthal, 2018).

Third, age disparities between groups may contribute to observed trajectory differences. FEP participants were older at baseline, and the age of psychosis onset itself could interact with cognitive aging processes. Later onset of first-episode psychosis might coincide with preexisting age-related neural changes, while earlier onset could disrupt neurodevelopmental milestones that influence long-term cognitive resilience (Zhang et al., 2025a). Additionally, the cumulative impact of untreated psychosis duration (even in medication-naïve participants) might accelerate cognitive decline in older FEP individuals (Sponheim et al., 2010), particularly in executive domains where age moderates the relationship between processing speed and task performance. Thuaire et al. (2022) showed that in schizophrenia, age exacerbates the effect of processing speed on cognitive shifting and updating, suggesting that older patients rely more heavily on intact processing speed to maintain executive function (Thuaire et al., 2022). Collectively, factors such as psychosis-induced functional disruption, baseline cognitive impairments, and intergroup age differences likely converge to generate the dissociative aging trajectories observed in individuals with FEP.

This study's strengths, including a relatively large sample size, comprehensive neurocognitive assessments, and a drug-naive cohort, provide robust evidence for the divergent age-related cognitive trajectories between FEP and HC. However, several limitations should be considered. First, the baseline age difference between the FEP and HC groups may confound the interpretation of age-related cognitive changes, potentially influencing the observed disparities in cognitive trajectories (Zhang et al., 2024b). Second, the absence of IQ assessment means that unmeasured intellectual factors could have affected cognitive performance and masked or exaggerated group differences. Third, as a cross-sectional study, it cannot establish causal relationships or accurately capture the longitudinal progression of cognitive decline in FEP and HC, limiting our understanding of the dynamic interplay between age and cognition (van Os, 2013; Zhang et al., 2023a, Zhang et al., 2025b). Finally, data collection from a single center may introduce selection bias and limit the generalizability of the findings, as the sample may not fully represent diverse populations with FEP or healthy individuals across different regions.

## 5 Conclusion

This study demonstrates that first-episode psychosis is associated with disrupted age-associated cognitive patterns, characterized by atypical patterns of decline and unexpected improvements in specific domains compared to healthy controls. These findings highlight the need for longitudinal research to further clarify the mechanisms underlying altered cognitive aging in psychosis and inform age-adapted intervention strategies.

## CRediT authorship contribution statement

**ChengFei Duan:** Writing – review & editing, Writing – original draft, Visualization, Resources, Data curation, Conceptualization. **ChunHua Cao:** Writing – review & editing, Writing – original draft, Resources, Data curation, Conceptualization. **MingLiang Ju:** Writing – review & editing, Writing – original draft, Validation, Investigation, Data curation, Conceptualization. **YanYan Wei:** Writing – review & editing, Methodology, Investigation. **XiaoChen Tang:** Writing – review & editing, Methodology, Formal analysis, Data curation. **LiHua Xu:** Writing – review & editing, Methodology, Investigation. **HuiRu Cui:** Writing – review & editing, Methodology, Investigation. **YingYing Tang:** Writing – review & editing, Validation, Software, Formal analysis, Data curation. **ZhengHui Yi:** Writing – review & editing, Supervision. **Xin Wei:** Writing – review & editing, Writing – original draft, Software, Resources, Data curation, Conceptualization. **JiJun Wang:** Writing – review & editing, Writing – original draft, Visualization, Validation, Supervision, Software, Resources, Conceptualization. **TianHong Zhang:** Writing – review & editing, Writing – original draft, Visualization, Validation, Supervision, Software, Methodology, Investigation, Funding acquisition, Formal analysis, Data curation, Conceptualization.

## Funding

This study was supported by the 10.13039/501100002855Ministry of Science and Technology of the People's Republic of China, 10.13039/501100012166National Key Research and Development Program of China (2023YFC2506800), 10.13039/501100001809National Natural Science Foundation of China (82171544, 82371505, 82151314, 82101623), The Shanghai Municipal Health Commission Clinical Research Special Project (202440203), and STI 2030-Major Projects (2022ZD0208500).

## Declaration of competing interest

The authors report no biomedical financial interests or potential conflicts of interest.
